# Standardized Uptake Value Using Thyroid Quantitative SPECT/CT for the Diagnosis and Evaluation of Graves' Disease: A Prospective Multicenter Study

**DOI:** 10.1155/2019/7589853

**Published:** 2019-01-30

**Authors:** Feng Dong, Lin Li, Yanzhu Bian, Guoquan Li, Xingmin Han, Mei Li, Juzhen Liu, Yong Xue, Yuhao Li, Yujing Hu, Jian Tan

**Affiliations:** ^1^Department of Nuclear Medicine, Tianjin Medical University General Hospital, Tianjin, China; ^2^Department of Nuclear Medicine, West China Hospital of Sichuan University, Chengdu, Sichuan, China; ^3^Department of Nuclear Medicine, Hebei General Hospital, Shijiazhuang, Hebei, China; ^4^Department of Nuclear Medicine, Xijing Hospital, The Fourth Military Medical University, Xi'an, Shanxi, China; ^5^Department of Nuclear Medicine, The First Affiliated Hospital of Zhengzhou University, Zhengzhou, Henan, China; ^6^Department of Nuclear Medicine, Beijing Tongren Hospital, Capital Medical University, Beijing, China; ^7^Department of Nuclear Medicine, The People's Hospital of Inner Mongolia Autonomous Region, Hohhot, Inner Mongolia, China; ^8^Department of Nuclear Medicine, Guangzhou Panyu Central Hospital, Guangzhou, China

## Abstract

The clinical applications of the quantitative single photon emission computed tomography (SPECT)/computed tomography (CT) are being expanded to a variety of fields of nuclear medicine. However, clinical application of quantitative SPECT/CT for the evaluation of Graves' disease (GD) still needs further investigation. Our aim was to investigate the feasibility of standard uptake value (SUV) of the thyroid for the clinical diagnosis and evaluation of GD. In this prospective multicenter study, 116 patients diagnosed with GD (Graves group) and 74 healthy volunteers (control group) were enrolled from 8 different hospitals. All patients underwent technetium pertechnetate (^99m^TcO_4_^−^) SPECT/CT imaging with Q.Metrix quantitative software and 24-hour thyroid radioactive iodine uptake (24h-RAIU) test. The SUVmax and SUVmean in Graves group were significantly higher than those of control group (*P*<0.01). Cut-off values of SUVmax and SUVmean to predict GD were 231.425 and 116.66 by ROC curves, respectively. The SUVmax and SUVmean in Graves patients were significantly related to serum thyroxine level with correlation coefficient of 0.493 and 0.512 for FT_3_ and 0.449 and 0.464 for FT_4_, respectively (all* P*<0.01). Additionally, the SUVmax and SUVmean in GD positively correlated with 24h-RAIU with a coefficient of 0.832 and 0.830, respectively (*P*<0.01). The volumes determined by Q.Metrix (35.65 ± 20.56ml) of 72 subjects also positively correlated with that from ultrasound (36.67 ± 21.00ml) with a coefficient of 0.927 (*P*<0.01). SUV measurements derived from thyroid SPECT/CT may be useful for the clinical diagnosis and evaluation of GD.

## 1. Introduction

Generally, in traditional nuclear medicine imaging, single photon emission computed tomography (SPECT) modality has been used for qualitative diagnosis by visual evaluation of radioactive distribution. For quantitative analysis, the target/nontarget (T/NT) ratio calculated from counts of their regions of interest (ROI) was widely used in SPECT modality, which was known as the semiquantitative analysis [1, 2]. Positron emission tomography (PET) was regarded as the only modality capable of absolute quantitative analysis in the past [3–5]. However, recently, owing to the development and application of a series of techniques including iterative reconstruction, scatter correction, CT attenuation correction, and resolution recovery, SPECT is also provided with the ability of absolute quantitation [6, 7]. Q.Metrix, a quantitative assessment software which employs SPECT and CT segmentation tools for quantifying radiopharmaceutical uptake, has been commercially available in SPECT/CT systems to measure standardized uptake value (SUV) using the same methods that are currently used to calculate SUV for PET images. Using a SPECT/CT scanner capable of quantitative imaging, absolute radioactivity concentration and SUV have been reported for ^99m^Tc-phosphonate bone SPECT/CT [8]. Furthermore, joint disease, such as temporomandibular disorder, has been successfully evaluated using the SUV derived from the quantitative bone SPECT/CT [9]. Indeed, the clinical applications of the quantitative SPECT/CT are being expanded to a variety of fields of nuclear medicine [10–13].

Graves' disease (GD) is the commonest cause of hyperthyroidism and accounted for nearly 90% cases [14, 15]. Technetium pertechnetate (^99m^TcO_4_^−^), a radioactive tracer used to assess thyroid function by thyroid uptake system, enters into thyroid follicular cells via a sodium-iodide symporter as does radioiodine, and the ^99m^TcO_4_^−^ thyroid scintigraphy is considered useful for the differential diagnosis between conditions characterized by extremely altered thyroid function, such as GD and destructive thyroiditis for nearly 5 decades in nuclear medicine [16–18]. Additionally, SUV based on thyroid parameters has been reported in the study by Lee et al. [12], which well compared the SUV using thyroid quantitative SPECT/CT with traditional ^99m^TcO_4_^−^  %thyroid uptake. However, clinical application of quantitative SPECT/CT for the evaluation of GD still needs further investigation.

In this prospective multicenter study, we used Q.Metrix to calculate SUV and thyroid volumes based on automatic segmentation on SPECT/CT images. SUV values were used to investigate quantitative differences between GD patients and healthy volunteers and their correlations with serum thyroxine level and 24h-RAIU; thyroid gland volumes calculated from Q.Metrix were compared to these measured from ultrasound. Generally, the diagnostic and potential evaluation values of quantitative measurements predicting GD were investigated.

## 2. Materials and Methods

This multicenter clinical trial was registered in clinical trials.gov (NCT02772705) and approved by the ethics committee of Chinese Academy of Medical Sciences and the institute review board of each participating center.

### 2.1. Subject Recruitment

From March 2016 to September 2016, 116 patients were diagnosed with GD and 74 healthy volunteers were recruited from 8 Chinese centers (Table 1) and then entered for the centralized analysis procedure. The need for written and informed consent was waived by each subject before study. All procedures performed in studies involving human participants were conducted in accordance with the Helsinki Declaration of 1975 (2000 revision).

The inclusion criteria for patients with GD were as follows: patients firstly diagnosed with definite clinical evidence, without taking or stopping antithyroid medicines at least one week prior scan and without taking exam of lipiodol radiography or any iodine agents within 1 month. Low iodine diet was required for the patients 1-2 weeks prior examination. The exclusion criteria included inflammatory hyperthyroidism, secondary hyperthyroidism due to drugs, HCG, and TSH-secreting pituitary tumor. The inclusion criteria for healthy volunteers: all 74 healthy volunteers were adults with normal laboratory thyroid function and also without a history of thyroid disease.

### 2.2. Measurements

For both GD patients and healthy subjects, serum FT_3_ (reference 3.50–6.50 pmol/L), FT_4_ (reference 11.50–23.50 pmol/L), and TSH (reference 0.30–5.00*μ*IU/mL) assays were tested on a fully automated ADVIA Centaur analyzer (Siemens Healthcare Diagnostics, New York, USA) by chemiluminescent reaction principle. Thyroid ultrasonography was performed on 72 subjects of patients who came from Tianjin Medical University General Hospital via an ultrasonic tester (Philips HD11) and the thyroid volume was estimated using the formula of a rotation ellipsoid. All GD patients underwent 24 h thyroid radioactive iodine uptake (RAIU) test. The thyroid 24h-RAIU value was measured 24 hours after an oral tracer dose (approximately 74 kBq) of ^131^I through a radioactive iodine uptake probe (MN-6300XT Apparatus, Technological University, China), which was calculated using the following equation: RAIU (%) = (neck counts – background counts) × 100/(standard counts – background counts).

### 2.3. SPECT/CT Acquisition Protocol

Firstly, the system planar sensitivity of SPECT camera in each center was measured according to the National Electrical Manufacturers Association (NEMA) guidelines, which measured the ratio of collimated counts rate detected in one acquisition plane to activity of a specific planar source placed parallel to the plane.

The ^99m^TcO_4_^−^ thyroid scintigraphy was performed by using a SPECT/CT scanner (NM/CT670; GE Healthcare, Pittsburgh, PA, USA) equipped with low-energy, high-resolution collimators. Regional planar images over anterior cervical region in the supine head-first position were obtained 30 minutes after the intravenous injection of 185MBq of ^99m^TcO_4_^−^. The ^99m^TcO_4_^−^  activity in the syringe before injection, the remnant ^99m^TcO_4_^−^  activity in the syringe after injection, and the measurement time were all recorded.

Immediately after the planar thyroid scintigraphy acquisition, quantitative SPECT/CT images were acquired by using the same SPECT/CT scanner. CT images were first obtained by using the following parameters: tube voltage of 120kV, tube current 80mA, rotating time 0.6s, table speed 13.75mm/rot, pitch 1.375:1, matrix size 512×512, and slice thickness of 3.75mm. No contrast agent was used. CT images were reconstructed using an adaptive statistical iterative reconstruction algorithm (ASiR, GE Healthcare) into 2.5 mm thick transaxial slices. Then, SPECT images were acquired by using the following parameters: photo peak and energy window 140±10% keV, scatter window 120±5% keV, matrix size 128×128, zoom factor 1.5, scan speed 20s/frame, and 6°/360°.

Additionally, SPECT images were reconstructed by using an iterative ordered subset expectation maximization (OSEM) algorithm (2 iterations and 10 subsets) with CT-based attenuation correction, scatter correction, and resolution recovery in the vendor supplied software (Q.Metrix; GE Healthcare). A postreconstruction filter (Butterworth with frequency of 0.48 and order of 10) was applied.

### 2.4. Parameters of the Quantitative SPECT/CT

**Figure 1:**
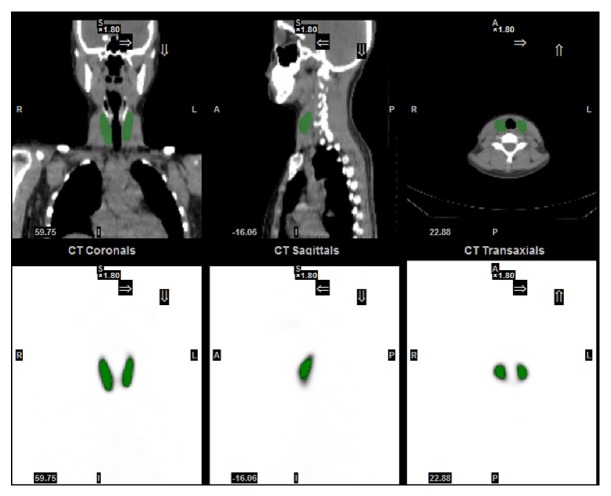
An illustration of VOI segmentation of thyroid using Q.Metrix. The VOIs of thyroid denoted by green colour were automatically segmented on the SPECT images for the SUV measurement with a NM threshold of 0.4.

Acquisition information was input into system including camera sensitivity, patient demographics, activities in full and empty syringe, administration time, scan time, and tracer information. Then the volumes of interest (VOI) of thyroid gland area were automatically segmented using NM threshold 0.4 on SPECT images (Figure 1). Both SUVmax and SUVmean values could be automatically calculated in the system using the following equation:

(1) SUVmeang/mL=Total  radioactivity/Volume  of  VOIInjected  radioactivity/Body  weightSUVmaxg/mL=Maximum  radioactivity/Volume  of  VoxelInjected  radioactivity/Body  weight

Note that the voxel volume for the SUVmax calculation was 3.2 × 10^−3^ mL in the present study.

### 2.5. Statistical Analysis

All numerical data are provided as the mean ± SD (standard deviation). Data were statistically analyzed using SPSS software (Version 20.0). Comparisons between Graves group and control group were performed using the Mann-Whitney* U* test due to nonnormal distributions. A chi square test was used to compare proportions between them. Optimal cut-off value was calculated by receiver operating characteristic (ROC) analysis. Correlations of SUVmean and SUVmax with serum thyroxine level and 24h-RAIU, as well as thyroid volume from quantitative SPECT images with that of ultrasound, were analyzed using the Spearman correlation analyses. A* P* value of <0.05 was considered statistically significant.

## 3. Results

### 3.1. Patient Characteristics

The 8 centers that participated in this multicenter study are listed in Table 1. Patient demographic and clinical characteristics of the Graves group and control group are shown in Table 2. There were no significant differences in gender and age between the 2 groups (*P*=0.99 and* P*=0.21, respectively), which included 116 cases of GD and 74 healthy volunteers. Additionally, patients with GD had higher levels of FT_3_ and FT_4_ compared with healthy volunteers (both* P* <0.01).

### 3.2. Comparisons of Quantitative Parameters of SUVmean and SUVmax between Groups

**Figure 2:**
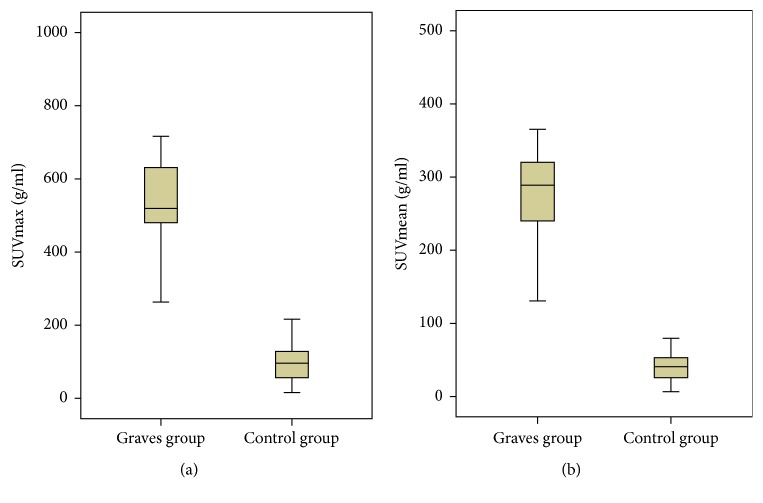
Comparison of SUVmax (a) and SUVmean (b) values between the 2 groups.

The SUV values of Graves group and control group are shown in Figure 2. The SUVmax and SUVmean values by quantitative SPECT/CT were significantly different between the 2 groups (both* P*<0.01, Mann-Whitney* U* test). Furthermore both SUVmax and SUVmean values of Graves group were significantly higher than those of control group.

### 3.3. Diagnostic Values of SUVmax and SUVmean for GD

**Figure 3:**
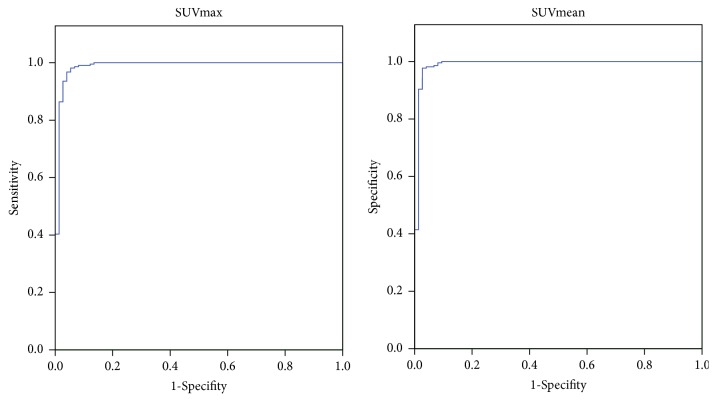
ROC curves of SUVmax (left) and SUVmean (right) in Graves group.

The ROC curves were drawn to evaluate the accuracy of SUVmax and SUVmean in predicting GD (Figure 3). The optimal cutoffs were the values yielding maximum sums of sensitivity and specificity from the ROC curves [19]. The results demonstrated that SUVmax had the area under the curve (AUC) of 0.995 (95% CI: 0.702, 0.926,* P *< 0.01), and the optimal cut-off value of SUVmax was 231.425, which yielded a sensitivity of 98.2% and a specificity of 95.9% for the detection and exclusion of GD, respectively. Similarly, we found a SUVmean threshold of 116.66, with a sensitivity of 98.2% and specificity of 97.3% for GD (AUC: 0.997; 95% CI: 0.714, 0.927,* P *< 0.01).

### 3.4. Correlations of Quantitative Parameters with Thyroid Hormone Levels and 24h-RAIU

**Figure 4:**
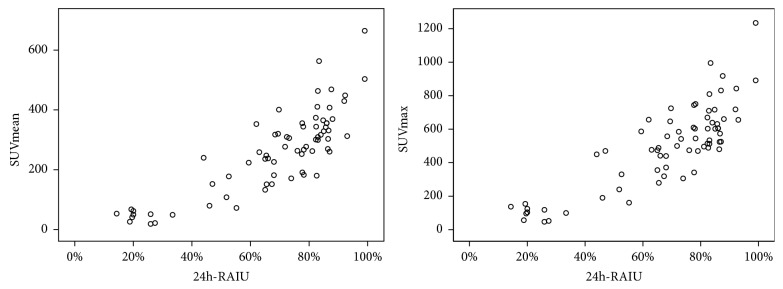
Correlation results between SUVmean (left), SUVmax (right), and 24h-RAIU in Graves group.

We investigated the correlations between SPECT/CT quantitative parameters and thyroid hormone levels. In the GD patients, both SUVmean and SUVmax had significant positive correlations with the FT_3_ and FT_4_ levels (*P*<0.01, r=0.512, 0.464 for SUVmean and 0.493, 0.449 for SUVmax, respectively). Similarly, 24h-RAIU also correlated positively with SUVmean and SUVmax (*P*<0.01, r=0.830 and 0.832, respectively) (as shown in Table 3 and Figure 4). Additionally, we found that Spearman coefficients between SUV values and 24h-RAIU were higher than those between SUV values and serum thyroxine (FT_3_ and FT_4_), which only ranged from 0.449 to 0.512.

However, in the control group, either SUVmean or SUVmax was not correlated with FT_3_, FT_4_, or 24h-RAIU (*P* = 0.831, 0.771, 0.859 for SUVmean and* P* = 0.761, 0.695, 0.917 for SUVmax, respectively).

### 3.5. Correlations of Thyroid Volume Measured by Quantitative Analysis and Ultrasound

**Figure 5:**
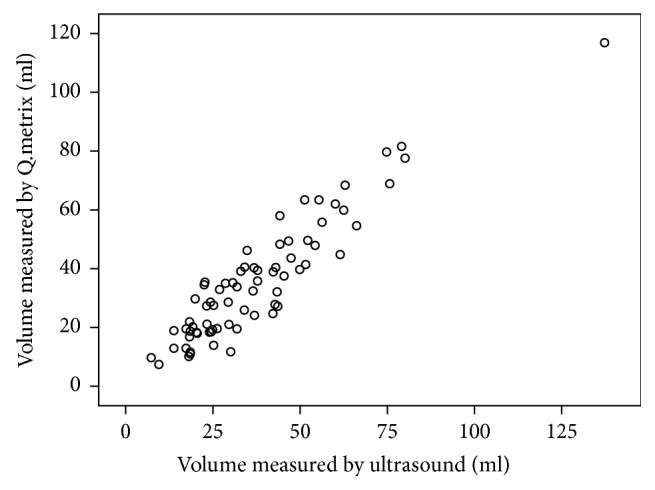
Correlation result between the volume obtained by Q.Metrix and ultrasound.

In 72 patients who previously underwent thyroid ultrasound examination, the average volume was 36.67 ± 21.00ml. The average thyroid volume measured on SPECT/CT images using Q.Metrix was 35.65 ± 20.56ml, which was consistent with that measured by ultrasound (*r*=0.927,* P*<0.01). The results were displayed in Figure 5.

## 4. Discussion

Quantitation has already been accepted for both anatomic imaging and PET imaging, and the SUV value of ^18^F-FDG PET/CT has widely been used as quantitative diagnostic criteria that reflects the FDG uptake ability in a target tissue [4]. However, PET/CT system has not broadly been used in many regions such as thyroid due to its high expense and inherent limitation [20]. Thanks to the development of tomographic imaging with a three-dimensional image reconstruction algorithm, planar nuclear imaging has evolved to SPECT. Despite the wide array of clinical applications in many fields of nuclear medicine, SPECT/CT has been considered not quantitative but qualitative, and the diagnostic results using SPECT are affected by many factors such as imaging parameters (for example, image background, noise, and contrast) skills and experiences of nuclear medicine doctors, which all lead to the limitation of diagnostic value of SPECT imaging. Recently, Q.Metrix, a quantitative assessment tool, has been commercially available in SPECT/CT systems to measure SUV values using the same methods such as attenuation compensation, scatter correction, and resolution recovery that are currently used for PET images. Using a quantitative SPECT/CT scanner, SUV values have been reported for ^99m^Tc-phosphonate bone SPECT/CT [8] and are being expanded to a variety of fields of nuclear medicine [10–13]. However, according to our literature search, the clinical application of quantitative SPECT/CT in the evaluation of GD still lacks.

### 4.1. Application of SUV Values in the Diagnosis of GD

Conventionally, the diagnosis of GD depends on not only clinical symptoms, but also examinations such as serum thyroxine levels, thyroid 131-iodine uptake test, and/or SPECT [21–23]. As for GD, the essential test is the serum FT_3_ and FT_4_ level, which reflects the function of thyroid more accurately than TT_3_ and TT_4_ [15]. In our study, we were trying to expand the quantitative application of SPECT/CT in the diagnosis of GD and observed that both SUVmax and SUVmean values derived from quantitative SPECT/CT images were positively correlated with FT_3_ and FT_4_ levels, which indicated that both SUVmax and SUVmean showed potential as useful parameters for the diagnosis and evaluation of GD. Furthermore, we found the cut-off values of SUVmax and SUVmean predicting GD were 231.425 and 116.66, respectively, based on ROC analysis, with satisfactory sensitivity and specificity.

### 4.2. SUV Values versus Thyroid 24h-RAIU

Conventionally, thyroid 131-iodine uptake test is the major quantitative method used in evaluating thyroid ability of iodine uptake, in making a distinction between hyperthyroidism and thyroiditis and predicting therapeutic dosage of 131-iodine in patients with GD [24]. However, it takes a relatively longer time (up to 24 hours required) than ^99m^TcO_4_^−^ thyroid scintigraphy, which takes only 15 to 30 minutes from injection to SPECT scan. Additionally, its quantitation is less precise as counts of thyroid-surrounding tissues such as oral cavity, parotid, and salivary gland are all included in the calculation of thyroid 131-iodine uptake which often leads to the overestimation of the thyroid iodine uptake [12]. Moreover, comparing to thyroid scintigraphy, less radioactivity (approximately 74 kBq) can be given into patients but with more radiation hazard to the thyroid, unenabling higher quality scintigraphic images.

In China, our guideline considers that thyroid 131-iodine uptake is at least 30% higher than that as indication of 131-iodine therapy to hyperthyroidism. For those patients, whose iodine uptake is around the 30% border, we wish to use SUV values in helping to screen proper subjects or determine reasonable therapeutic dosage based on the actual ability of iodine uptake. Furthermore, there are few researches on the relativity of thyroid quantitative technetium uptake and iodine uptake due to lack of reliable quantitative measurement in conventional SPECT. In the present study, we found that both SUVmean and SUVmax in Graves group had significant positive correlations with 24h-RAIU, which indicated the potential application of SUV values in therapeutic guidance.

### 4.3. Thyroid Volume

Currently, in China, thyroid volume can be measured by either ultrasound or SPECT imaging. The result measured via ultrasound is affected by the experience of the operator. SPECT, as a functional imaging, could better reflect the functional tissue than ultrasound. However, due to the limitation of planar imaging, the results obtained by these two methods vary greatly. In this study, we found that volumes obtained using Q.Metrix were significantly relevant to those measured by ultrasound with a correlation coefficient of 0.927. With the application of autosegmented volume and SUV measurements in SPECT/CT, both the diagnosis and evaluation of GD, which contribute to the ^131^I dose determination, could be achieved at the same time.

Since our study is a multicenter study, in order to ensure the consistency, the same model SPECT/CT scanner and the same imaging protocol were applied to all the measurements in the 8 centers, and the interscanner calibration was validated by measuring the system planar sensitivity for each center ahead of acquisition. The mean sensitivity was 143.22±3.21 counts/min/*μ*Ci.

However, there are several limitations in the present study. Firstly, the sample is not large enough. A larger scale of research is expected in the future. Secondly, in this study we did not recruit the thyroiditis patients, who have moderate or decreased thyroid 131-iodine uptake but maybe high serum thyroxine level; therefore a thyroiditis group will be enrolled in the future work to improve the study design. Furthermore, since this study was conducted in a country with rich iodine-intake, the results may not be the same in low iodine-intake countries.

## 5. Conclusion

In summary, SUV measurements derived from thyroid SPECT/CT using Q.Metrix were applicable in clinic and useful for the clinical diagnosis and evaluation of GD, with very high sensitivity and specificity.

## Figures and Tables

**Table 1:** Recruited patient number of 8 participant centers.

Centers	Patient number	Healthy volunteer number	Total
Tianjin Medical University General Hospital	48	38	86
West China Hospital of Sichuan University	14	33	47
Hebei General Hospital	25	2	27
Xijing Hospital	12	1	13
The First Affiliated Hospital of Zhengzhou University	8	0	8
Beijing Tongren Hospital	4	0	4
The People's Hospital of Inner Mongolia Autonomous Region	4	0	4
Guangzhou Panyu central hospital	1	0	1

Total	116	74	190

**Table 2:** Comparison of patient demographics and serum thyroxine level between Graves group and control group.

	Graves group	Control group	*P* Value
	(n=116)	(n=74)	
Gender (Female : Male)	83 : 33	53 : 21	0.992
Age	35.5 ± 11.9	38.3 ± 19	0.213
FT_3_	25.3 ± 14.8	4.75 ± 0.75	**<0.01**
FT_4_	63.7 ± 34.3	16.7 ± 2.65	**<0.01**

Data are presented as mean ± SD

**Table 3:** Correlations of SUV values with FT_3_, FT_4_ and 24-h thyroid iodine uptake in Graves group.

	FT_3_	FT_4_	24-h thyroid iodine uptake
SUV Mean			
*r* value	0.512	0.464	0.830
*P *value	<0.01	<0.01	<0.01
SUV Max			
*r* value	0.493	0.449	0.832
*P* value	<0.01	<0.01	<0.01

## Data Availability

The data used to support the findings of this study are available from the corresponding author upon request.
